# Idiopathic scrotal calcinosis: Is the cause still unknown? A case report

**DOI:** 10.1016/j.ijscr.2024.109503

**Published:** 2024-03-11

**Authors:** Wael Gazzah, Souheil Ben Taher, Rayen Lahouar, Badreddine Ben Khalifa, Sahbi Naouar, Braiek Salem

**Affiliations:** University of Sousse, Department of Urology, Ibn El Jazzar Hospital, Kairouan, Tunisia

**Keywords:** Scrotum, Calcinosis cutis, Epidermal cyst, Case reports, Urology

## Abstract

**Introduction and importance:**

Idiopathic Scrotal Calcinosis (ISC) is a rare and benign dermatological condition, characterized by the formation of calcified nodules on the scrotal skin.

**Case presentation:**

A 47-year-old man with a 15-year history of painless, chamois-colored nodules on his scrotum. Surgical excision of the affected skin was performed, followed by primary closure, with histopathological examination confirming ISC. The patient recovered well with no recurrence noted at a 14-month follow-up.

**Clinical discussion:**

The etiology of ISC remains uncertain, with theories ranging from dystrophic calcification of epidermal cysts to Dartos muscle degeneration. In this case, no signs of epithelial cells or anatomical structure degeneration were observed, supporting the idiopathic nature of ISC. Treatment is typically surgical and aimed at aesthetic or symptomatic relief. While surgery is generally effective, the literature indicates a variable risk of recurrence, underscoring the need for long-term follow-up.

**Conclusion:**

This report contributes to the understanding of ISC, highlighting its idiopathic nature and the diversity of its etiological theories. It reinforces the effectiveness of surgical treatment for symptomatic relief and underscores the importance of ongoing research to elucidate the condition's etiology and optimize patient care.

## Highlights

• Idiopathic Scrotal Calcinosis: rare dermatological condition in scrotal skin
• Effective surgical treatment for aesthetic and symptomatic relief
• Case study of a 47-year-old patient with ISC
• No recurrence noted at 14-month post-operative follow-up
• Highlights idiopathic nature and diverse etiological theories of ISC

## 1 Introduction

Idiopathic Scrotal Calcinosis (ISC) is a rare, benign condition that affects the scrotal skin, and is characterized by single or multiple calcified nodules that vary in size and number. First reported in 1883, this condition was named by Shapiro almost a century later [1].

ISC typically occurs in children and young adults and is not associated with any disorders of the calcium‑phosphorus metabolism [2]. In certain cases, the entire scrotal skin may appear to be calcified. Although it might be assumed that sophisticated reconstruction techniques are necessary after excision, primary closure is often sufficient, as demonstrated in this study.

Histologically, ISC is characterized by calcium deposits of varying sizes, surrounded by a granulomatous reaction. The pathophysiology of ISC remains a topic of considerable debate.

In this article, we present a case of ISC outlining surgical treatment and provide a review of the relevant literature.

## 2 Presentation of case

**Fig. 1:**
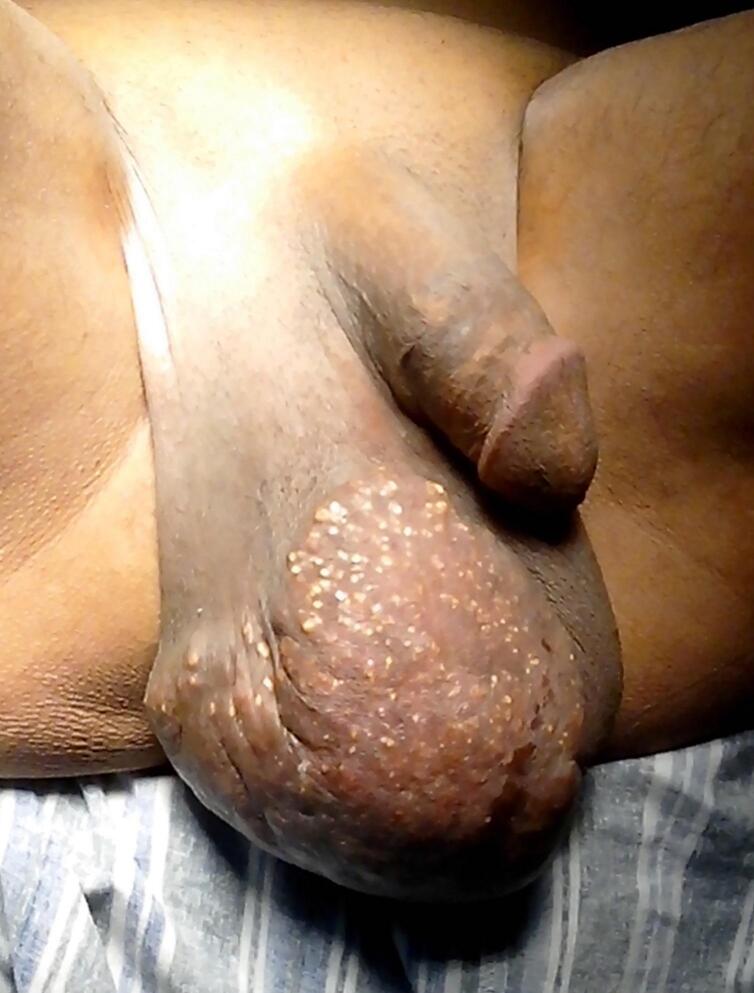
Multiple painless nodules involving the scrotum.

A 47-year-old man presented to our department with scrotal skin lesions (Fig. 1). He first noticed nodules on his scrotum 15 years prior to the consultation, which gradually increased in size and number. Physical examination revealed numerous painless, chamois-colored subcutaneous nodules on the scrotal skin. The patient had no history of metabolic, endocrine, neoplastic or autoimmune disorders. All biological assessments, including the phospho‑calcium balance and bacteriological analyses, were normal.

**Fig. 2:**
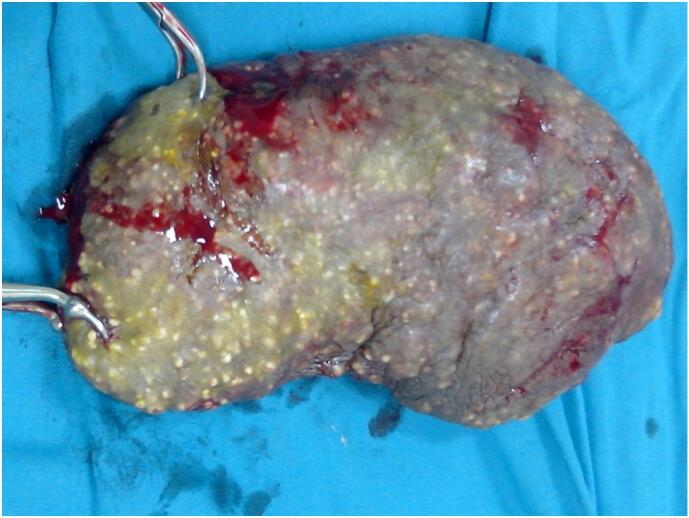
Excised scrotal skin.

**Fig. 3:**
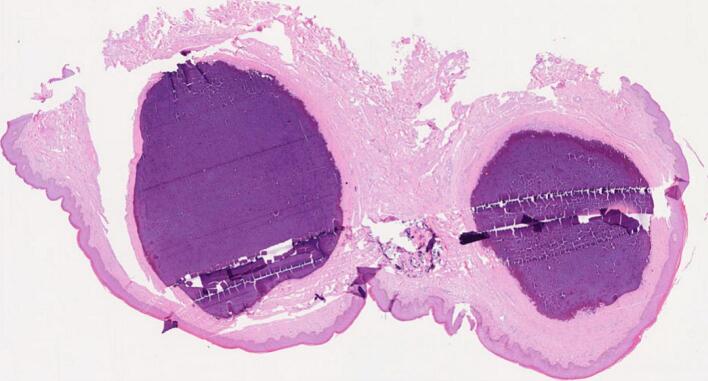
Histopathologic view of specimen (hematoxylineosin stain, original magnification x10).

**Fig. 4:**
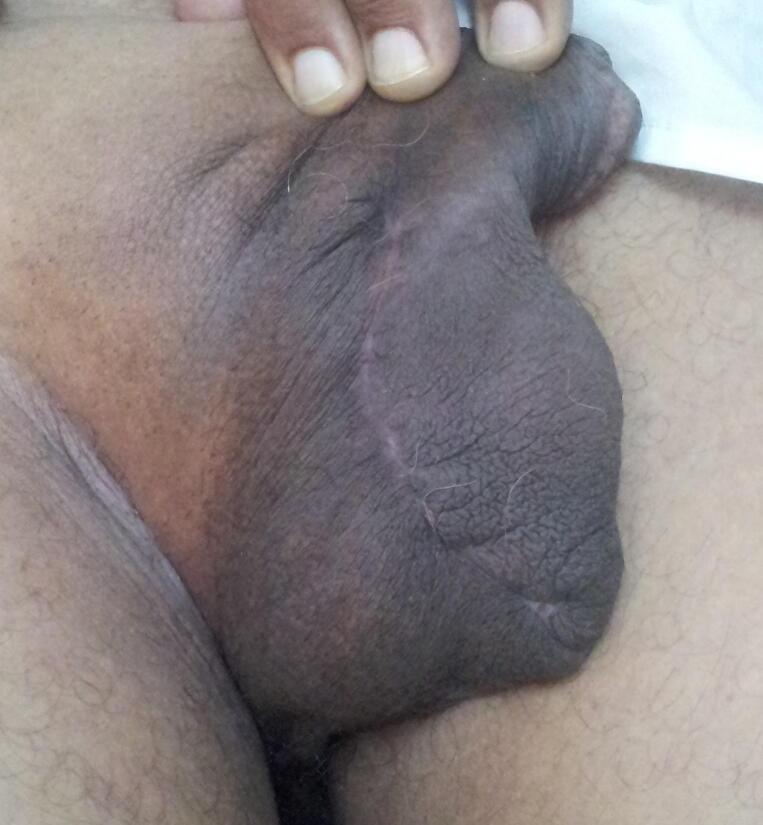
Photo at 3 months follow up.

Under spinal anesthesia, the affected scrotal skin was excised above the Dartos fascia, followed by closure of the remaining skin (Fig. 2). The patient recovered uneventfully postoperatively. Histological examination of the dermal connective tissue showed large foci of calcifications (Fig. 3), occasionally accompanied by granulomatous inflammatory foci and dilated hair follicles. Anatomopathological findings confirmed the diagnosis of scrotal calcinosis. A 14-month follow-up revealed no recurrence of the nodules (Fig. 4).

## 3 Discussion

Idiopathic Scrotal Calcinosis (ISC) is a benign, rare entity first described in 1883 by Lewinski. It primarily affects men aged 20–40 years [3]. Clinically, ISC manifests as one or more nodules on the scrotal skin, which are yellow to brown in color and grow slowly. These nodules, ranging from 1 mm to several centimeters in diameter, are often asymptomatic, but may cause itching. Tsai et al. reported a case of ISC associated with chronic pelvic and perineal pain [4].

The etiology of ISC is not fully understood. Wright et al. studied 9 patients and concluded that the condition was idiopathic [5]. Some studies have suggested that ISC originates from dystrophic calcification of epidermal cysts, as evidenced by epidermal cells found around calcified areas [6]. Saad and Zataari reported three ISC cases and concluded that epidermal cysts significantly contribute to the disease's pathogenesis [7].

Swinhart and Golitz hypothesized that calcification of epidermal cysts occurs following an inflammatory reaction, leading to a degenerative process and eventual epithelial lining loss [8]. Song et al. described the disease's progression in relation to nodules by examining 51 excised nodules from a single patient [9]. Progression includes cyst formation, calcification of intracystic material, wall weakening due to growth, rupture, mononuclear inflammation due to exposed intracystic content, and resorption of the cyst wall with its keratin content, leaving only calcified material.

King et al. supported Dartos muscle degeneration theory [10]. However, this has not been universally accepted. While our case demonstrated ISC confined to the dermis layer, allowing for effective treatment through skin excision while preserving the Dartos, it is important to consider varying presentations of ISC. Notably, King et al. observed histopathological findings of calcified foci within the Dartos layer, suggesting a deeper involvement in some cases. This indicates that surgical approaches might need individual tailoring based on the specific histopathological characteristics of each case. Our findings support the need for careful preoperative evaluation and consideration of varied treatment strategies in ISC, acknowledging that some cases may involve deeper tissue layers, as shown in other studies. Versess and Malik and Feinstein et al. suggested that minor trauma may initiate the ISC process [11,12].

Microscopic examination in our case showed no signs of epithelial cell or anatomical structure degeneration, leaving the etiology idiopathic. ISC is benign, and treatment is recommended only for aesthetic or symptomatic relief. Surgical excision should be confined to the scrotal skin as calcified nodules reside in the dermis. Extensive scrotal calcinosis can make post-excision defects challenging to manage.

However, the risk of recurrence remains controversial. Some researchers believe that surgery is an effective solution, whereas others point to a high recurrence probability of ISC [13]. In our patient, the surgical outcome was satisfactory without postoperative complications, although the follow-up period was too short to definitively assess the adequacy of treatment.

## 4 Conclusion

Idiopathic Scrotal Calcinosis is a rare condition with a poorly understood etiology. Our case study, along with a literature review, underscores the idiopathic nature of ISC and the diversity of its etiological theories. Surgical treatment has proven effective for aesthetic or symptomatic reasons, as demonstrated in our patient, with no postoperative complications or short-term recurrence. However, the potential for recurrence highlighted in the literature suggests the importance of long-term follow-ups. This study contributes to a better understanding of ISC and highlights the need for future studies to deepen our knowledge and improve patient care.

## Informed consent

Written informed consent was obtained from the patient for the publication of this case report and any accompanying images. A copy of the written consent form is available for review by the Editor-in-Chief of this journal upon request.

## SCARE guidelines

This study was reported in line with the SCARE criteria [14].

## Ethical approval

This study is exempt from ethical approval as per the policies of Ibn El Jazzar Hospital.

## Funding

No funding was received for conducting this study.

## Guarantor

Wael Gazzah.

## CRediT authorship contribution statement

All authors have contributed equally to the work reported in this manuscript, including the conception, design, execution, data acquisition, analysis and interpretation, and the drafting and revising of the manuscript for important intellectual content.

## Declaration of competing interest

The authors declare that they have no conflicts of interest concerning this article.
